# Subtype-specific prognostic implications of plasma-detected *PIK3CA* mutations in Vietnamese breast cancer patients

**DOI:** 10.3389/fonc.2025.1663823

**Published:** 2025-12-03

**Authors:** Dinh Thi Thao, Dong Van Quyen, Le Huu Song, Ngo Tat Trung

**Affiliations:** 1 Center for Genetic Consultation and Cancer Screening, 108 Military Center Hospital, Hanoi, Vietnam; 2 University of Science and Technology of Hanoi, Hanoi, Vietnam; 3 Institute of Biotechnology, Vietnam Academy of Science and Technology, Hanoi, Vietnam; 4 Vietnamese-German Center for Medical Research, 108 Military Center Hospital, Hanoi, Vietnam; 5 Department of Molecular Biology, Laboratory Center, 108 Military Central Hospital, Hanoi, Vietnam

**Keywords:** breast cancer, circulating tumor DNA, cell-free DNA, mutation analysis, liquid biopsy, PIK3CA mutation, prognostic biomarker

## Abstract

**Background:**

*PIK3CA* mutations are among the most frequent genomic alterations in breast cancer (BC), contributing to disease progression and therapeutic resistance. Non-invasive blood assays can reveal tumor-specific DNA alterations, enhancing personalized oncology.

**Aim:**

This study aims to investigate the clinical relevance of plasma-detected *PIK3CA* mutations in Vietnamese breast cancer patients, with a focus on subtype-specific outcomes.

**Methods:**

*PIK3CA* hotspot mutations (H1047R and E545K) were detected in plasma from 196 BC patients. Associations with clinicopathological features and progression-free survival (PFS) were assessed.

**Results:**

*PIK3CA* mutations were identified in 42.9% of patients with H1047R (31.6%) more prevalent than E545K (15.3%). Mutation rates were highest in HR+ subtypes and elevated in advanced or irradiated patients (p = 0.009). E545K was enriched in HR+ cases, while H1047R was more frequent in HER2+ tumors following radiotherapy. Among metastatic BC patients, those with *PIK3CA* mutations had shorter PFS (median, 7.0 vs. 15.0 months; p = 0.022), and univariate Cox regression showed increased progression risk (HR = 2.16), although not significant after multivariate adjustment. E545K was associated with lung (p = 0.047) and bone metastases (p = 0.012) and H1047R was enriched in brain metastases (p = 0.028).

**Conclusion:**

Plasma-detected *PIK3CA* mutations, particularly E545K and H1047R, exhibited subtype-specific associations with clinical outcomes, indicating that plasma analysis may provide complementary information for prognostic assessment in metastatic BC.

## 1 Introduction

Breast cancer (BC) is a molecularly heterogeneous disease and remains the most frequently diagnosed malignancy among women globally. According to the Global Cancer Observatory (GLOBOCAN 2022), BC accounted for over 2.3 million new cases, with a growing burden in low- and middle-income countries, including Vietnam (1). Despite advances in early detection and targeted therapies, disease recurrence, metastasis, and treatment resistance remain major challenges in clinical management. Among molecular subtypes, hormone receptor-positive (HR+), HER2-negative (HER2-) BC accounts for the majority of cases and is primarily treated with endocrine therapy. Despite initial responsiveness, roughly 20% of patients experience disease progression or recurrence or distant metastasis due to acquired resistance to endocrine agents (2). Aberrant activation of the phosphoinositide 3-kinase (PI3K)/Akt/mTOR signaling pathway has been recognized as a key mechanism implicated in this treatment resistance (3).

Somatic mutations in the *PIK3CA* gene, which encodes the p110α catalytic subunit of the PI3Kα complex, are the most frequent genetic alterations observed in HR+/HER2− BC, occurring in approximately 20% to 40% of patients. These mutations, particularly those at hotspots in exon 9 (E545K) and exon 20 (H1047R), result in constitutive pathway activation and contribute to tumor progression, and therapeutic resistance (3). While the prognostic and predictive roles of *PIK3CA* mutations have been explored in various populations, data remain limited and inconsistent, particularly in underrepresented cohorts. The mutations have been correlated with improved prognosis in early-stage BC (4, 5); however, emerging evidence suggests an association between these mutations and reduced treatment efficacy, including resistance to endocrine and chemotherapeutic therapies, as well as unfavorable outcomes (6–12). Conversely, several studies have failed to demonstrate a consistent predictive or prognostic role for *PIK3CA* mutations (13–16).

Recent advances in targeted therapy have led to the development of PI3Kα inhibitors such as alpelisib and inavolisib, which have shown clinical benefit and are approved for the treatment of *PIK3CA*-mutated HR+/HER2− advanced BC when combined with fulvestrant (17, 18). Furthermore, capivasertib, an AKT inhibitor, has demonstrated efficacy in HR+/HER2− BC patients harboring *PIK3CA*, *AKT1*, or *PTEN* alterations following disease progression on standard adjuvant therapies (19). In recent years, liquid biopsy techniques, particularly plasma-derived circulating tumor DNA (ctDNA) analysis, have been known as a minimally invasive approach for detecting relevant mutations and guiding treatment decisions in real time. Liquid biopsy is particularly advantageous in patients with inaccessible metastatic lesions or for longitudinal monitoring of tumor evolution (20). Consequently, *PIK3CA* mutation testing via liquid biopsy is increasingly recommended when evaluating patients who are candidates for PI3K-targeted therapies or AKT inhibitors, especially in cases of endocrine-resistant disease (21).

Most prior investigations have focused on tissue-based profiling, which may not fully capture spatial and temporal tumor heterogeneity, particularly in the metastatic setting. The increasing clinical adoption of circulating tumor DNA (ctDNA) analysis provides a minimally invasive approach to real-time tumor monitoring; however, the clinical relevance of plasma-detected *PIK3CA* mutations—especially in relation to specific molecular subtypes and treatment outcomes—remains insufficiently characterized. Moreover, despite emerging evidence suggesting distinct biological behaviors and therapeutic implications of individual *PIK3CA* variants, such as E545K and H1047R, comparative analyses of their prognostic impact remain limited. These gaps underscore the need for more detailed, variant-specific investigations using liquid biopsy–based approaches to inform personalized management strategies in BC.

In this research, we utilized a recently developed blocker-mediated asymmetric PCR assay (22) designed to investigate the frequency and subtype-specific prognostic implications of *PIK3CA* mutations (H1047R and E545K) in plasma-derived cell-free DNA (cfDNA) from BC patients. This study aims to evaluate the association between plasma *PIK3CA* mutation status and clinical outcomes, with the goal of clarifying its potential relevance as a prognostic biomarker and contribution to individualized treatment strategies.

## 2 Materials and methods

### 2.1 Patients and sample collection

A total of 196 patients diagnosed with BC were enrolled at the 108 Military Central Hospital (MCH), Vietnam, between June 2021 and June 2023. All participants provided written informed consent prior to inclusion in the study. Patient information was completely anonymous. All procedures were approved by the institutional ethics and scientific committees. The study protocol received ethical approval from the Medical Ethics Committee of the 108 MCH, Hanoi, Vietnam (Aprroval number: 2527/21-5-2021). Venous blood samples and relevant clinical data were collected at the time of enrollment.

Progression-free survival (PFS) was defined as the duration from the date of blood collection to the first confirmed local recurrence or distant metastasis, based on the RECIST guideline (23). Disease progression was assessed by the attending physicians using clinical and/or radiographic evidence, and findings were documented in patients’ medical records.

### 2.2 Sample preparation

Whole blood was drawn into EDTA tubes and centrifuged at 2,000 × g for 10 minutes at room temperature within 2 hours of collection. Plasma was separated, aliquoted, and stored at −80 °C. cfDNA was extracted from 500 μL of plasma using the MagMAX™ Cell-Free DNA Isolation Kit (Thermo Fisher Scientific, Waltham, MA, USA), following the manufacturer’s instructions. The extracted cfDNA was stored at −80 °C until further use.

### 2.3 Control samples: positive and negative genomic DNA

Breast cancer cell lines MCF7 and T-47D (Thermo Fisher Scientific, USA) were cultured in RPMI 1640 medium (Invitrogen, Carlsbad, CA, USA) supplemented with 7.5% fetal bovine serum and 100 U/mL penicillin-streptomycin (Sigma-Aldrich, USA), and maintained at 37 °C in a humidified atmosphere with 5% CO_2_;. Genomic DNA was isolated from MCF7, T-47D, and peripheral white blood cells of healthy donors using the Genomic DNA Purification Kit (Thermo Fisher Scientific), with elution in 100 μL of buffer. Extracted DNA was aliquoted and stored at −20 °C. To generate sample controls, MCF7 cells (carrying ~30% E545K mutant allele) or T-47D cells (carrying ~50% H1047R mutant allele) were mixed with healthy donor cells to achieve mutant allele frequencies of 10% and 0%. DNA extracted from these mixtures was used as a positive control (10%) and a negative control (0%) during cfDNA analysis.

### 2.4 Detection of *PIK3CA* mutations using asymmetric PCR

Blocker-mediated asymmetric PCR was used to detect *PIK3CA* hotspot mutations (H1047R and E545K). Reactions were performed on the LightCycler 96 real-time PCR system (Roche Diagnostics, Mannheim, Germany). The thermal cycling protocol included: initial denaturation at 95 °C for 10 minutes, followed by 50 cycles at 95 °C for 15 seconds, 55 °C for 20 seconds, and 72 °C for 20 seconds.

Each 20 μL reaction mixture contained 10 μL of 2× Universal PCR Master Mix (no UNG; Applied Biosystems, Foster City, CA, USA), 0.8 μL each of forward and reverse primers, 1.6–2.4 μL of allele-specific blocker oligonucleotides, and 4 μL of cfDNA template. SYBR Green fluorescence was monitored in real time, and data were analyzed using the accompanying software.

### 2.5 Oligonucleotides and PCR reagents

Primers and blockers were designed based on recommendations from a previous study (24). Detailed sequences of the primers and blockers, as well as the limit of detection values of the asymmetric PCR assays, are provided in the **Supplementary Materials** (**Supplementary Table S1**). All oligonucleotides were synthesized by Integrated DNA Technologies (IDT, Coralville, IA, USA). Additional reagents, including the PCR master mix, nuclease-free water, deoxynucleotide triphosphates (dNTPs), and loading buffer, were obtained from Thermo Fisher Scientific (Waltham, MA, USA).

### 2.6 Immunohistochemical analysis

Pathological assessment was performed by certified pathologists at the Department of Pathology, Laboratory Center, 108 MCH. Estrogen receptor (ER) and progesterone receptor (PR) expression was assessed via immunohistochemistry (IHC), with ≥1% nuclear staining considered positive (25). HER2 status was determined by IHC or confirmed by fluorescence *in situ* hybridization (FISH) for equivocal (2+) cases. A HER2 IHC score of 3+ or a positive FISH result was considered HER2-positive (26).

### 2.7 Statistical analysis

Statistical analyses were performed using IBM SPSS Statistics version 25.0 (IBM Corp., Armonk, NY, USA). Associations between *PIK3CA* mutations and clinicopathological characteristics were evaluated using the chi-square test or Fisher’s exact test as appropriate. PFS was estimated using the Kaplan–Meier method, and differences between groups were assessed using the log-rank test. The median follow-up duration was 24.0 months (95% CI, 22.7–25.3). Univariate and multivariate Cox regression analyses estimated Hazard ratios (HRs) and 95% confidence intervals (CIs) for PFS, adjusting for relevant clinical covariates. A p-value< 0.05 was considered statistically significant. No correction for multiple comparisons (e.g., Bonferroni or false discovery rate) was applied given the exploratory nature of the study and limited sample size; therefore, all subgroup analyses were interpreted as hypothesis-generating. Graphs were generated using Microsoft Excel 2010.

## 3 Results

### 3.1 Circulating *PIK3CA* mutations in breast cancer patients

**Figure 1:**
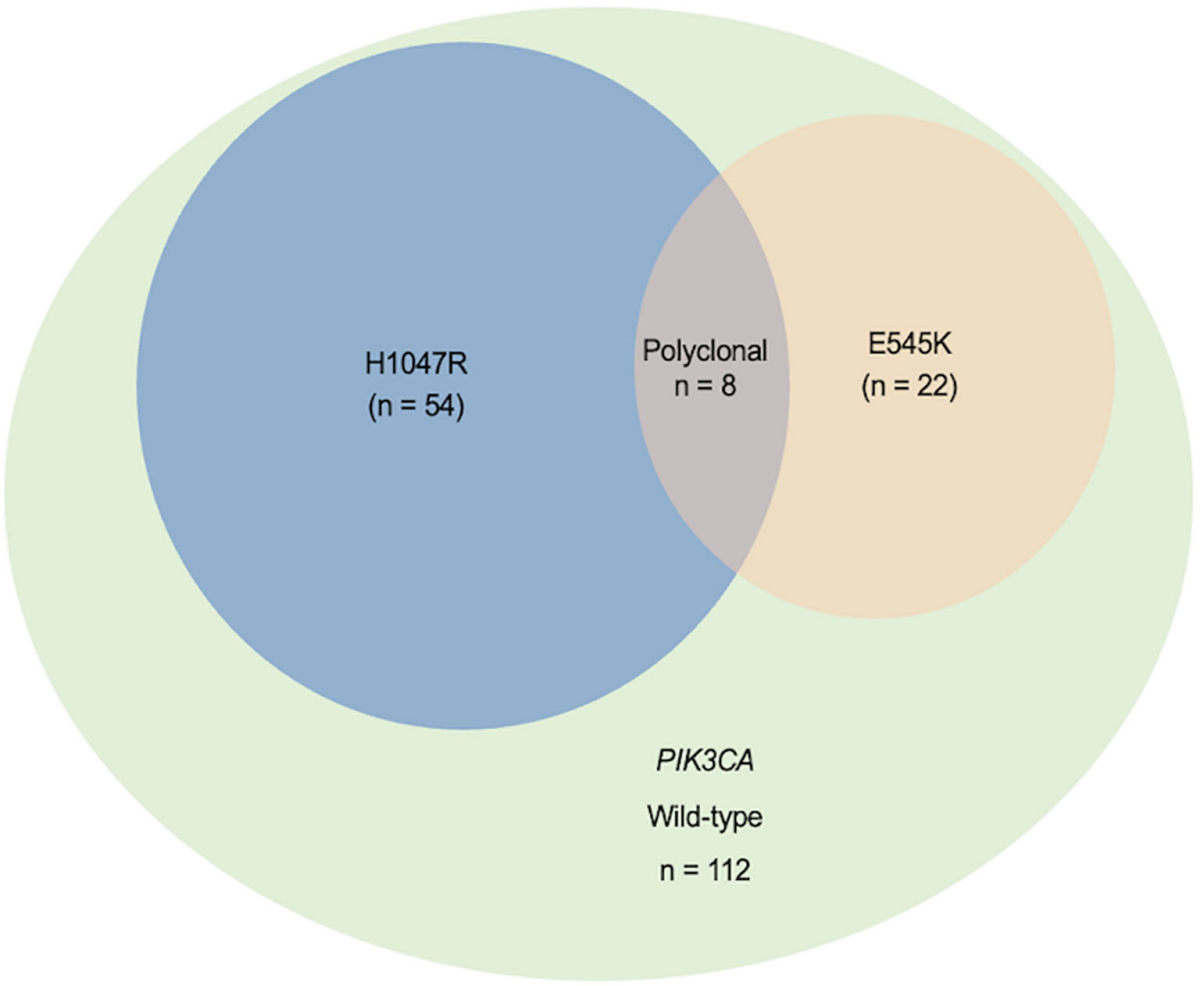
Distribution of plasma-detected *PIK3CA* hotspot mutations (H1047R and E545K) in 196 breast cancer patients. The Venn diagram displays the distribution of *PIK3CA* hotspot mutations (H1047R and E545K) and their overlap, indicating multiple mutant variants in plasma from 196 breast cancer patients.

**Figure 2:**
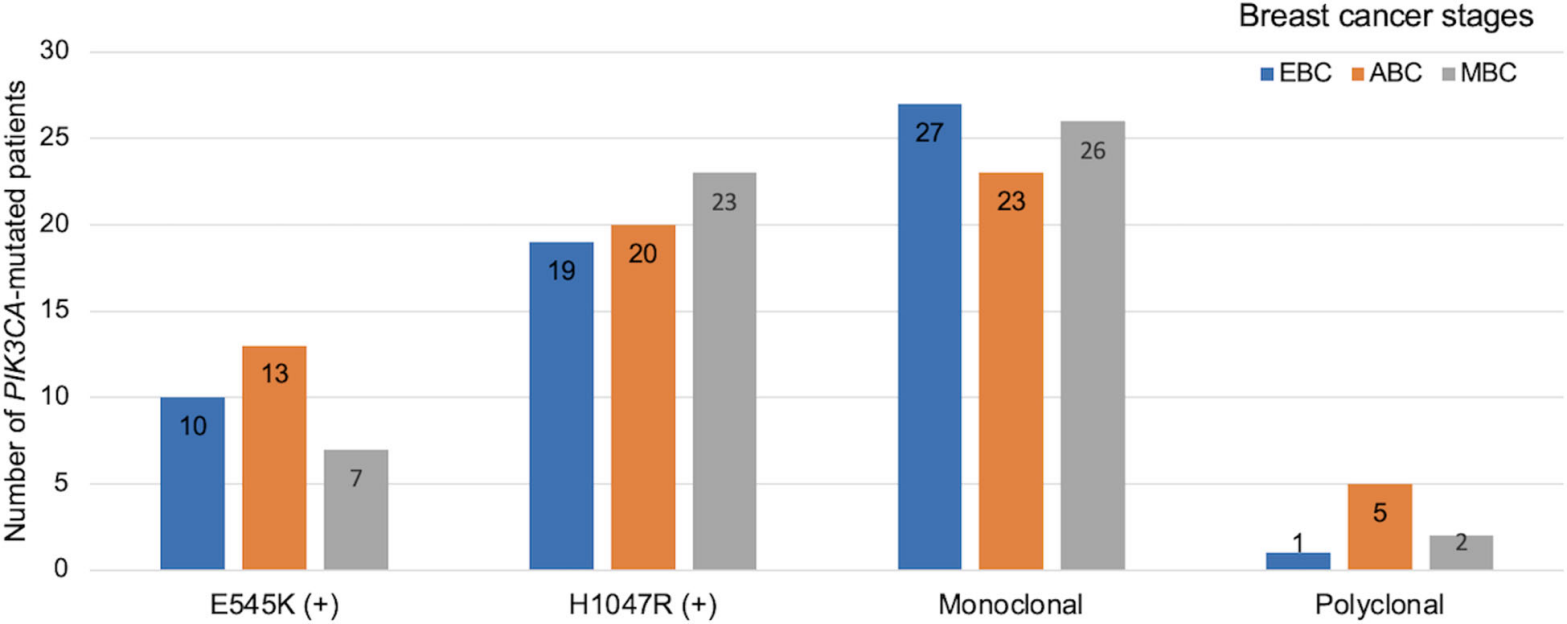
Distribution of *PIK3CA* mutation profiles and clonality across breast cancer stages. Bar chart shows the distribution of *PIK3CA* hotspot mutations and clonality patterns across clinical stages of breast cancer. EBC: early breast cancer (stage I–II, blue), ABC: advanced breast cancer (stage III, orange), MBC: metastatic breast cancer (stage IV, grey). Mutation subtypes include E545K and H1047R. Clonality status was categorized as monoclonal or polyclonal based on mutation patterns detected.

Using a blocker-mediated asymmetric PCR assay, we analyzed plasma samples from 196 BC patients. Circulating hotspot mutations in the *PIK3CA* gene (E545K and/or H1047R) were detected in 84 patients (42.9%). Notably, 8 patients (4.1%) harbored both mutations concurrently, while 112 patients (57.1%) tested negative for both (**Figure 1**). The H1047R mutation was more frequently observed than E545K, and most patients carried only one hotspot mutation regardless of the disease stages (**Figures 1**, **2**). Mutation frequencies differed across molecular subtypes. The HR+/HER2+ group had the highest prevalence (48.8%), followed by HR+/HER2− (45.2%), HR−/HER2+ (32.4%), and triple-negative (21.4%) subtypes (**Table 1**).

**Table 1:** *PIK3CA* mutation spectrum stratified by molecular subtypes of breast cancer (n= 196).

Breast cancer subtypes, n(%)	*PIK3CA* (+) 84 (42.9%)	E545K (+) 22 (11.2%)	H1047R (+) 54 (27.6%)	Dual mutations 8 (4.1%)
HR+/HER2- (n= 62)	28 (45.2%)	12 (19.4%)	17 (27.4%)	1 (1.6%)
HR+/HER2+ (n = 86)	42 (48.8%)	13 (15.1%)	34 (39.5%)	5 (5.8%)
HR-/HER2+ (n= 34)	11 (32.4%)	5 (14.7%)	8 (23.5%)	2 (5.9%)
Triple negative (n=14)	3 (21.4%)	0 (0%)	3 (21.4%)	0 (0%)

*PIK3CA*/E545K/H1047R (+): mutation positive; HER2: human epidermal growth factor receptor-2; HR: hormone receptor.

Of the total cohort, 43 patients (21.9%) experienced disease recurrence, 54 (27.6%) were classified as stage IV, and 116 (59.2%) presented with at least one site of invasion-either nodal or distant metastases (**Table 2**). The prevalence of *PIK3CA* mutations significantly increased with disease stage: 4.8% in stage I, 28.6% in stage II, 33.3% in stage III, and 33.3% in stage IV (p = 0.024). No statistically significant associations were observed between *PIK3CA* mutation status and age, menopausal status, or tumor histopathology (**Table 2**). However, mutation rates were significantly higher in patients with metastatic disease (p = 0.032), particularly in those with multiple metastatic sites (p = 0.032).

**Table:** Table 2 Characteristics of the study population according to circulating *PIK3CA* mutation status.

Variables	Total n = 196 (100%)	*PIK3CA* mutant status		P-value^#^
		Positive n = 84 (42.9%)	Negative n = 112 (57.1%)	
Age ( $\bar{X}$ ± SD, years)	52.43 ± 12.38	50.45 ± 12.46	53.92 ± 12.16	0.052
Disease stages				
I	18 (9.2)	4 (4.8)	14 (12.5)	**0.024**
II	71 (36.2)	24 (28.6)	47 (42.0)
III	53 (27)	28 (33.3)	25 (22.3)
IV	54 (27.6)	28 (33.3)	26 (23.2)
Tumor histology				
Ductal	191 (97.4)	83 (98.8)	109 (96.4)	0.394
Lobular	5 (2.6)	1.0 (1.2)	4 (3.6)
Grade				
1	12 (6.1)	3.0 (3.6)	9 (8.0)	0.395
2	108 (55.1)	49 (58.3)	59 (52.7)
3	76 (38.8)	32 (38.1)	44 (39.3)
Menopausal status				
Post-menopausal	109 (55.6)	41 (48.8)	68 (60.7)	0.111
Recurrence				
Yes	43 (21.9)	24 (28.6)	19 (17.0)	0.052
No	153 (78.1)	60 (71.4)	93 (83.0)	
Metastatic disease				
Yes	116 (59.2)	57 (67.9)	59 (52.7)	**0.032**
No	80 (40.8)	27 (32.1)	53 (47.3)
The number of metastasis lesion				
0	80 (40.8)	27 (32.1)	53 (47.3)	**0.032**
≤ 2	94 (48)	43 (51.2)	51 (45.5)
≥ 3	22 (11.2)	14 (16.7)	8 (7.1)
Metastatic sites				
Lymph nodes	104 (53.1)	50 (59.5)	54 (48.2)	0.148
Viscera	34 (17.3)	19 (22.6)	15 (13.4)	0.091
Bone	29 (14.8)	17 (20.2)	12 (10.7)	0.063
Treatment				
Surgical therapy	161 (82.1)	49 (79)	112 (83.6)	0.431
Hormone therapy	98 (50)	43 (51.2)	55 (49.1)	0.885
Chemotherapy	175 (89.3)	78 (92.9)	97 (86.6)	0.243
Radiotherapy	60 (30.6)	34 (40.5)	26 (23.2)	**0.009**

Statistically significant values (p< 0.05) are shown in bold; p-value^#^ Mutation versus wild-type; HER2: human epidermal growth factor receptor-2; HR: hormone receptor.

**Figure 3:**
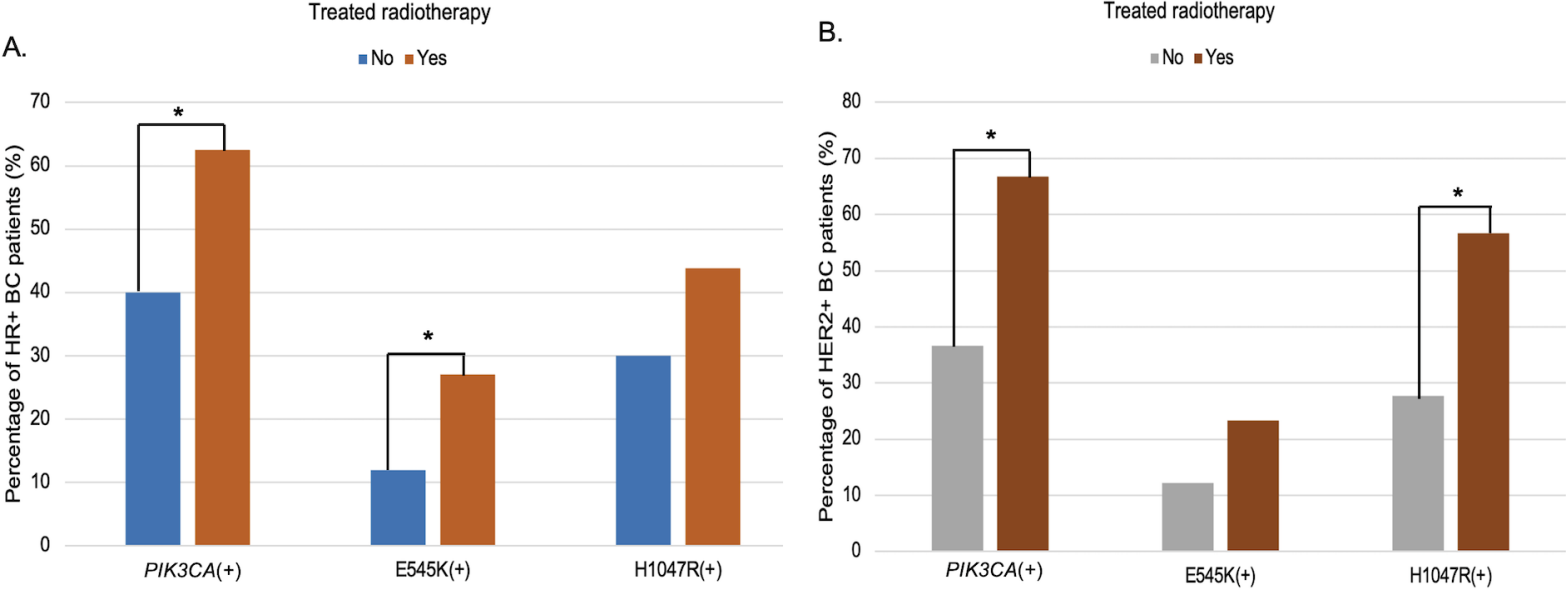
Subtype-specific association of *PIK3CA* mutations with radiotherapy among HR+ **(A)** and HER2+ **(B)** breast cancer. Bar charts depict the distribution of circulating *PIK3CA*, E545K, and H1047R mutations according to radiotherapy exposure, stratified by receptor subtype. **(A)** In HR+ BC patients, the frequency of *PIK3CA* and E545K mutations was significantly higher in those who received radiotherapy compared to those who did not (*p* < 0.05). **(B)** Among HER2+ BC patients, *PIK3CA* and H1047R mutations were significantly more common in the radiotherapy group (*p* < 0.05). “*”: statistically significant differences (*p* < 0.05).

Furthermore, *PIK3CA* mutations were significantly associated with patients who had received radiotherapy (p = 0.009) (**Table 2**). Stratified analysis revealed mutation subtype-specific enrichment in distinct treatment subgroups: the E545K variant was more frequent in radiotherapy-treated HR+ BC patients (OR = 2.72; 95% CI, 1.13–6.55; p = 0.022), while the H1047R mutation was enriched in HER2+ BC patients who had undergone radiotherapy (OR = 3.45; 95% CI, 1.47–8.13; p = 0.004; **Figure 3**, **Supplementary Table S2**).

### 3.2 Association between plasma-detected *PIK3CA* mutations and prognosis in metastatic breast cancer

**Figure 4:**
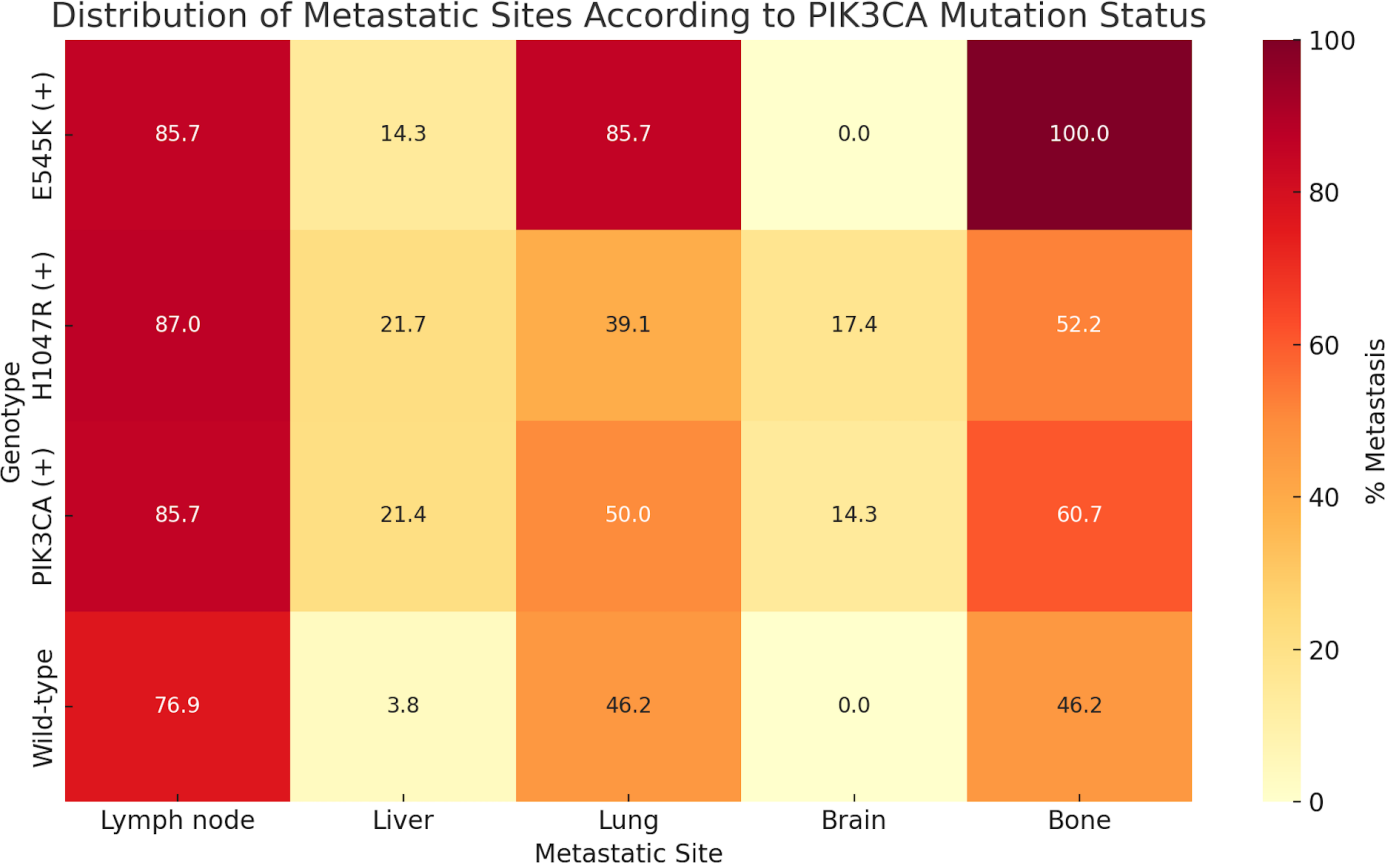
Distribution of metastatic sites according to plasma-detected *PIK3CA* mutation status. The percentage of patients with metastases to specific organs—including lymph nodes, liver, lung, brain, and bone—is shown for each genetic subgroup: E545K-positive (E545K (+)), H1047R-positive (H1047R (+)), any *PIK3CA* mutation-positive (*PIK3CA* (+)), and wild-type. Notably, E545K mutations were associated with higher rates of lung (85.7%) and bone (100%) metastases, whereas H1047R mutations were more frequently detected in patients with brain metastases (17.4%). Wild-type patients exhibited lower rates of visceral and bone metastases compared to those with *PIK3CA* mutations.

A focused analysis of the 54 patients with metastatic BC (detailed characteristics presented in **Supplementary Tables S3**, **S4**) revealed no significant association between overall *PIK3CA* mutation status and metastatic distribution. However, variant-specific analysis demonstrated that the E545K mutation was significantly associated with lung (OR = 8.1; 95% CI, 0.9–71.1; p = 0.047) and bone metastases (OR = 17.0; 95% CI, 0.9–315.6; p = 0.012), while H1047R mutations were more frequently observed in patients with brain metastases (OR = 14.5; 95% CI, 0.7–285.1; p = 0.028; **Figure 4**, **Supplementary Table S5**).

**Figure 5:**
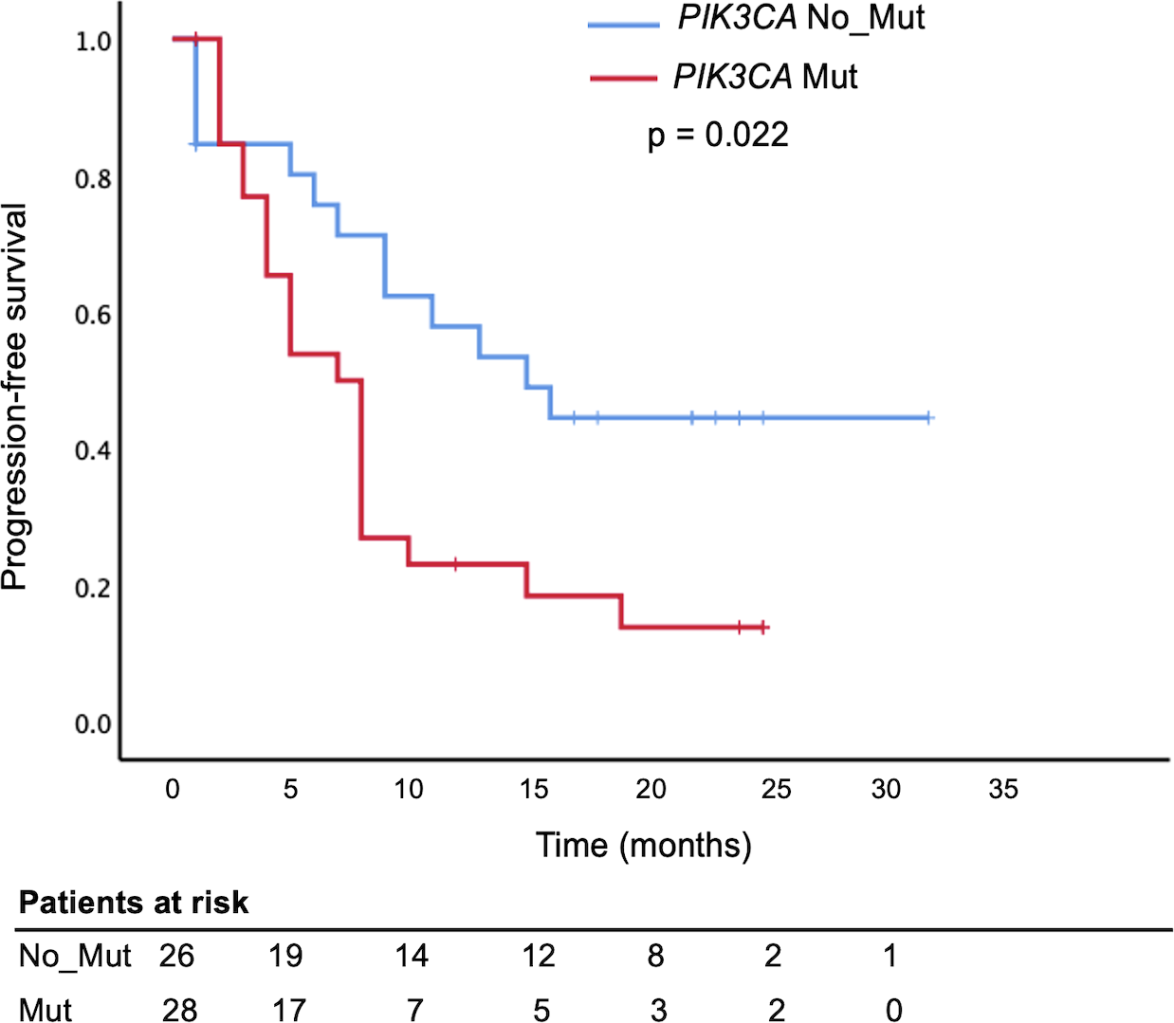
Shorter Progression-Free Survival in metastatic breast cancer patients with circulating *PIK3CA* mutations. Kaplan–Meier survival analysis comparing progression-free survival (PFS) in metastatic breast cancer patients based on plasma *PIK3CA* mutation status. Patients with detectable *PIK3CA* mutations in circulating cell-free DNA (*PIK3CA* Mut, red curve) had significantly shorter PFS compared to those without mutations (*PIK3CA* No_Mut or wild-type, blue curve), log-rank test, p = 0.022.

Furthermore, there was a significant association between *PIK3CA* mutations and disease progression (OR = 3.67; 95% CI, 1.10 – 12.0; p = 0.028; **Table 3**). PFS analysis using the Kaplan–Meier method and log-rank test demonstrated that metastatic BC patients harboring *PIK3CA* mutations had significantly shorter PFS than those without (median, 7 months; 95% CI, 5.0–9.0 vs. 15 months; 95% CI, 7.35–22.64; p = 0.022). In contrast, no significant difference in PFS was observed when patients were stratified by individual mutation subtype (E545K or H1047R; **Figure 5**, **Supplementary Table S6**). In univariate Cox regression, *PIK3CA* mutation was associated with an increased risk of progression (HR = 2.16; 95% CI, 1.07–4.35; p = 0.031); however, this association did not remain statistically significant after multivariate adjustment (HR = 1.58; 95% CI, 0.73–3.43; p = 0.245; **Table 4**).

**Table 3:** Association between *PIK3CA* mutation status and disease progression in the metastatic breast cancer patients (n = 54).

Mutation		Progression		p-value	OR (95%CI)
		Yes	No		
*PIK3CA (overall)*	MT	22 (78.6%)	6 (21.4%)	**0.028**	3.67 (1.10- 12.0)
	WT	13 (50%)	13 (50%)
E545K	MT	6 (85.7%)	1 (14.3%)	0.40^*^	3.72 (0.40 - 33.5)
	WT	29 (61.7%)	18 (38.3%)
H1047R	MT	18 (78.3%)	5 (21.7%)	0.075	2.96 (0.87 – 10.0)
WT	17 (54.8%)	14 (45.2%)

OR, odds ratio; “*”, p-values obtained by Fisher’s exact test (two-sided). Statistically significant value (p< 0.05) is shown in bold. WT: wild-type, MT: mutation.

**Table 4:** Univariate and multivariate Cox proportional hazards regression analysis for progression-free survival in the metastatic breast cancer cohort (n = 54).

Variables	Univariate analysis		Multivariate analysis	
	HR (95%CI)	p-value	HR (95%CI)	p-value
*PIK3CA* mutation (mutant vs. wild-type)	2.16 (1.07 – 4.35)	**0.031**	1.58 (0.73 – 3.43)	0.245
Age (≤ 50 vs. > 50 years)	–	–	0.95 (0.41 – 2.18)	0.895
HER2 status (positive vs. negative)	–	–	0.86 (0.41 – 1.85)	0.709
Metastatic number (< 2 vs. ≥ 2)	–	–	0.55 (0.14 – 2.15)	0.387
Lymph node invasion (Yes vs. No)	–	–	1.58 (0.47 – 5.27)	0.458
Visceral invasion (Yes vs. No)	–	–	3.59 (1.36 – 9.51)	**0.010**
Bone invasion (Yes vs. No)	–	–	1.94 (1.79 – 4.77)	0.150

HR, hazard ratios; HER2, human epidermal growth factor receptor-2;

Statistically significant values (p< 0.05) are shown in bold.

## 4 Discussion

This study investigated the prevalence and potential prognostic significance of plasma-detected *PIK3CA* hotspot mutations (E545K and H1047R) across distinct molecular subtypes of BC in a Vietnamese cohort. These two variants are among the most predominant *PIK3CA* hotspots, together accounting for approximately 60–70% of all clinically relevant mutations reported in BC (3, 9, 10, 15, 17, 21). Our findings suggest the clinical utility of liquid biopsy in capturing tumor-derived genetic alterations and indicate that *PIK3CA* mutations are not uniformly distributed across subtypes, nor do they confer identical prognostic implications.

Consistent with prior literature, the overall frequency of plasma-detected *PIK3CA* mutations in our cohort (42.9%) falls within previously reported ranges (30% to 50%) based on tissue or liquid biopsy analyses in Western and Asian populations (3–5, 9). Notably, H1047R mutation was more prevalent than E545K, and co-occurrence of both mutations was rare, corroborating the mutually exclusive nature of most *PIK3CA* alterations (9–11, 15, 16). At the subtype level, the high prevalence of *PIK3CA* mutations in HR+/HER2− and HR+/HER2+ subtypes of our cohort aligns with prior studies (6, 13). Slight differences in mutation frequency across studies may reflect variations in assay sensitivity, sample types (tissue vs. plasma), and underlying population-specific genetic heterogeneity (3–6, 9, 10).

Beyond overall prevalence, our data also revealed an enrichment of *PIK3CA* mutations among patients who had received radiotherapy, suggesting a possible interplay between PI3K pathway activation and radiation sensitivity. Mechanistically, activation of the PI3K/Akt/mTOR pathway has been implicated in radioresistance, primarily through enhanced DNA repair and inhibition of apoptosis (27). Conversely, in some clinical cohorts, tumors harboring *PIK3CA* mutations have shown improved local control and longer recurrence-free survival following radiotherapy (28, 29). This inconsistency underscores the need for prospective, subtype-specific studies to clarify the predictive role of *PIK3CA* mutations in radiotherapy response.

Moreover, our study highlights the subtype-specific prognostic relevance of circulating *PIK3CA* mutations in the metastatic setting. In particular, the presence of the E545K mutation was significantly associated with lung and bone metastases, while H1047R was linked to brain metastasis. These trends, which are comparable to previous clinical observations (30), suggest that distinct mutations may influence could be linked to differences in metastatic tropism through variations in downstream PI3K/AKT/mTOR signaling or tumor–microenvironment interactions. At the molecular level, the helical-domain E545K and kinase-domain H1047R mutations activate the PI3K/AKT/mTOR pathway through distinct mechanisms—RAS-dependent and p85-dependent, respectively (13, 14, 31). These mechanistic differences previously demonstrated in experimental studies may help contextualize the mutation–site-related metastatic patterns observed in our cohort.

Importantly, *PIK3CA* mutations were significantly associated with disease progression and shorter PFS, but this effect was not retained after multivariate adjustment, suggesting that their prognostic impact may be confounded by tumor subtype and related prognostic covariates. Although previous studies have also reported correlations between *PIK3CA* status and patient outcomes (5–12, 14), multivariate analyses were often not clearly described, limiting definitive interpretation. Collectively, the prognostic significance of *PIK3CA* mutations remains uncertain and warrants validation in larger, well-controlled studies.

Building on these data, cfDNA testing enables minimally invasive detection of *PIK3CA* mutation patterns across BC subtypes, providing molecular insights that may refine prognostic evaluation and guide individualized treatment. In Vietnam, where access to NGS-based genotyping remains limited, cfDNA PCR-based testing offers a feasible complementary approach for identifying actionable alterations and assessing therapeutic eligibility, thereby facilitating the clinical application of targeted therapies in resource-limited settings. In this context, the blocker-mediated asymmetric PCR used in our study offers a practical option with acceptable analytical sensitivity (limit of detection 0.01–0.1%; **Supplementary Table S1**), reasonable cost, and broad instrument availability, making it potentially applicable for cfDNA analysis in routine laboratories.

The lack of matched plasma–tumor comparisons limits direct validation of cfDNA testing as a surrogate. Even so, previous studies have shown high concordance between plasma- and tissue-based genotyping in BC (32–34), and plasma–based *PIK3CA* testing is now recognized as a clinically supported option when tumor tissue is unavailable (21). Nonetheless, further validation in prospectively paired plasma–tumor cohorts remains necessary.

In addition, several other limitations should be acknowledged. First, our cohort was recruited from a single tertiary center, and detailed treatment data were not consistently available, which together may not fully capture the genetic diversity among Vietnamese BC patients. Second, the relatively small number of patients with metastatic disease may reduce the robustness of subgroup analyses. Third, potential competing risks, such as treatment crossover or non–cancer-related death, were not accounted for in this analysis and may have influenced time-to-event estimates. Accordingly, the findings should be interpreted with caution as exploratory and hypothesis-generating.

Despite certain limitations, this study provides comprehensive evidence on plasma-detected *PIK3CA* mutations in Vietnamese BC and supports the integration of liquid biopsy into clinical management for mutation profiling, risk stratification, and treatment selection.

## 5 Conclusion

Our data suggest that *PIK3CA* mutations are frequent in Vietnamese BC patients and show a trend toward prognostic relevance depending on molecular subtypes and mutation variants. Plasma-based detection represents a feasible approach for assessing tumor heterogeneity and may assist in predicting disease progression in the metastatic setting. Future prospective studies are warranted to validate these findings and to explore the predictive utility of *PIK3CA* mutations in guiding personalized therapies.

## Data Availability

The datasets presented in this study can be found in online repositories. The names of the repository/repositories and accession number(s) can be found in the article/**Supplementary Material**.
