# Supplementary material for: Subtype-specific prognostic implications of plasma-detected PIK3CA mutations in Vietnamese breast cancer patients
Source: Front Oncol. 2025 Dec 3;15:1663823. doi: 10.3389/fonc.2025.1663823 (PMC12708320; doi:10.3389/fonc.2025.1663823)
Supplement: Supplementary file 1 [file DataSheet1.docx]

**Supplementary Materials**

**Table S1.** Sequences of primers and blockers used in asymmetric PCR for detection of *PIK3CA* hotspot mutations

| ***PIK3CA* mutation** | **Primers and blocker** | **Sequences (5’-3’)** | **LOD**  **value** | **Reference** |
| --- | --- | --- | --- | --- |
| H1047R | F_ primer  R_ primer  WT_ BL | ACAAATGAATGATGCACG  CAGTTCAATGCATGCTGTTTAATT  TGATGCACATCATGG TG/PO4 | 0.01% | [22] |
| E545K | F_ primer  R_ primer  WT_BL | CCTCTCTCTGAAATCACGA  CTGAGATCAGCCAAATTCAGTTA  TGAAATCACTGAGCAGGAG/PO4 | 0.1% |  |
| Abbreviations: LOD: limit of detection, F: forward, R: reverse, WT_BL: wild-type-specific blocker. | | | | |

**Table S2.** Association between radiotherapy and plasma *PIK3CA* mutation status stratified by breast cancer subgroups

| **Mutation** | **Radiotherapy** | **HR+ BC**  **(n = 148)** | | **HER2+ BC**  **(n = 121)** | |
| --- | --- | --- | --- | --- | --- |
|  |  | **OR (95%CI)** | **p-value** | **OR (95%CI)** | **p-value** |
| *PIK3CA* (overall) | Yes vs. No | 2.50 (1.23 – 5.08) | **0.010** | 3.52 (1.47 – 8.40) | **0.004** |
| E545K | Yes vs. No | 2.72 (1.13 – 6.55) | **0.022** | 2.21 (0.77 – 6.36) | 0.133 |
| H1047R | Yes vs. No | 1.82 (0.89 – 3.70) | 0.099 | 3.45 (1.47 – 8.13) | **0.004** |
| Abbreviations: OR, odds ratio; HR: hormone receptor; HER2: human epidermal growth factor receptor-2; BC: breast cancer; Statistically significant values (p < 0.05) are shown in bold. | | | | | |

**Table S3.** Characteristics of metastatic BC patients with detected*-PIK3CA* mutation status

| **Variables** | **Total n = 54 (100%)** |
| --- | --- |
| Age ($\bar{X}$±SD, years) | 53.15 ± 11.68 |
| HR-positive | 40 (74.1) |
| HER2-positive | 31 (57.4) |
| Recurrence | 39 (72.2) |
| Progression | 35 (64.8) |
| **Number of metastatic locations** | |
| 1 | 13 (24.1) |
| ≥2 | 41 (75.9) |
| **Metastatic sites** | |
| Lymph node | 44 (81.5) |
| Bone | 29 (53.7) |
| Lung | 26 (48.1) |
| Liver | 7 (13) |
| Brain | 4 (7.4) |
| **Received therapy** | |
| Endocrine therapy | 39 (72.2) |
| Chemotherapy | 47 (87) |
| Radiotherapy | 23 (42.6) |
| **Gene mutant status** | |
| Detected *PIK3CA* gene mutations | 28 (51.9%) |
| H1047R mutation | 23 (42.6%) |
| E545K mutation | 7 (13%) |
| Abbreviations: HER2: human epidermal growth factor receptor-2; HR: hormone receptor | |

**Table S4.** Detailed clinical outcomes according to plasma *PIK3CA* mutation status in metastatic breast cancer

| **Patient ID** | ***PIK3CA***  **mutation** | **BC subtype** | **Progression**  **sites** | **PFS**  **(month)** |
| --- | --- | --- | --- | --- |
| No.1 | H1047R | HR+/HER2+ | Live, LN | 3 |
| No. 2 | H1047R | HR+/HER2+ | N/A | 1 |
| No. 3 | H1047R | HR-/HER2+ | Lung | 8 |
| No. 4 | H1047R | TN | N/A | 12 |
| No. 5 | H1047R | HR+/HER2+ | No | 25 |
| No. 6 | H1047R | HR+/HER2+ | Bone | 7 |
| No. 7 | E545K | HR+/HER2+ | N/A | 1 |
| No. 8 | H1047R | HR+/HER2- | Lung | 4 |
| No. 9 | H1047R | HR+/HER2+ | Live | 8 |
| No. 10 | H1047R | HR+/HER2+ | LN | 8 |
| No. 11 | E545K | HR+/HER2- | Bone | 8 |
| No. 12 | H1047R | HR+/HER2- | Live, bone | 15 |
| No. 13 | H1047R | HR-/HER2+ | Brain | 2 |
| No. 14 | H1047R | HR+/HER2- | Bone | 8 |
| No. 15 | H1047R | HR+/HER2+ | No | 25 |
| No. 16 | H1047R | HR+/HER2- | Bone | 4 |
| No. 17 | E545K | HR+/HER2+ | Bone | 2 |
| No. 18 | E545K | HR+/HER2- | Bone | 2 |
| No. 19 | E545K, H1047R | HR+/HER2+ | Bone | 8 |
| No. 20 | H1047R | HR-/HER2+ | Bone | 5 |
| No. 21 | H1047R | HR+/HER2- | LN | 2 |
| No. 22 | H1047R | HR+/HER2+ | Brain, bone | 5 |
| No. 23 | E545K | HR-/HER2+ | Bone | 19 |
| No. 24 | H1047R | HR+/HER2+ | Brain, bone | 3 |
| No. 25 | E545K, H1047R | HR+/HER2+ | LN | 4 |
| No. 26 | H1047R | TN | Live | 5 |
| No. 27 | H1047R | HR+/HER2- | Live | 10 |
| No. 28 | H1047R | HR+/HER2- | No | 24 |
| Abbreviations: PFS: progression-free survival; LN: lymph node; N/A: not available; No: no progression; HR: hormone receptor; HER2: human epidermal growth factor receptor-2; TN: triple negative | | | | |

**Table S5.** Association between *PIK3CA* hotspot mutations and metastatic sites among patients with metastatic breast cancer.

| **Metastatic site** | **E545K** | | **H1047R** | | ***PIK3CA*** | |
| --- | --- | --- | --- | --- | --- | --- |
|  | OR (95%CI) | p-value | OR (95%CI) | p-value | OR (95%CI) | p-value |
| Lymph node | 1.42  (0.15-13.33) | 1.000 | 1.94  (0.44-8.52) | 0.489 | 1.8  (0.45-7.28) | 0.494 |
| Live | 1.14  (0.12-11.18) | 1.000 | 4.03  (0.71-22.99) | 0.122^*^ | 6.82  (0.76-61.12) | 0.102^*^ |
| Lung | 8.10  (0.90-71.10) | **0.047^*^** | 0.53  (0.18-1.58) | 0.284 | 1.17  (0.40-3.40) | 0.793 |
| Bone | 17.0  (0.92-315.6)^#^ | **0.012^*^** | 0.90  (0.31-2.65) | 1.000 | 1.80  (0.61-5.32) | 0.413 |
| Brain | 0.64  (0.03-13.24)^#^ | 1.000^*^ | 14.5  (0.74-285.1)^#^ | **0.028^*^** | 2.08  (0.36-11.92) | 0.112^*^ |
| Abbreviations: WT: wild-type, MT: mutation, OR, odds ratio; “*”, p-values obtained by Fisher’s exact test (two-sided), statistically significant value (p < 0.05) is shown in bold. “#”: adjusted using the Haldane–Anscombe correction (+0.5) due to the presence of zero cell counts. | | | | | | |

**Table S6.** Association between *PIK3CA* mutations and progression-free duration among patients with metastatic breast cancer.

| ***PIK3CA* mutation status** | | **Progression-free duration (month)**  **Median (95% CI)** | **p-value** |
| --- | --- | --- | --- |
| *PIK3CA*  (overall) | Positive (n = 28) | 7 (5.0 – 9.0) | **0.022** |
|  | Negative (n = 26) | 15 (7.35 – 22.64) |  |
| H1047R | Positive (n = 23) | 7 (4.96 – 9.04) | 0.085 |
|  | Negative (n = 31) | 13 (4.28 – 21.72) |  |
| E545K | Positive (n = 7) | 4 (0 – 8.8) | 0.104 |
|  | Negative (n = 47) | 9 (6.27 – 11.72) |  |
| Abbreviations: Statistically significant values (p < 0.05) are shown in bold | | | |
